# Crosslinking and functionalization of acellular patches via the self-assembly of copper@tea polyphenol nanoparticles

**DOI:** 10.1093/rb/rbac030

**Published:** 2022-05-18

**Authors:** Qin Li, Yuan Gao, Jiajun Zhang, Yangfeng Tang, Yangyong Sun, Lujia Wu, Hao Wu, Meifang Shen, Xiaohong Liu, Lin Han, Zhiyun Xu

**Affiliations:** 1 Department of Cardiovascular Lab, Institute of Cardiothoracic Surgery, Changhai Hospital, Shanghai, China; 2 Institute of Cardiovascular Surgery, Changhai Hospital, Shanghai, China

**Keywords:** acellular patches, crosslinking, functionalization, self-assembly

## Abstract

Decellularization is a promising technique to produce natural scaffolds for tissue engineering applications. However, non-crosslinked natural scaffolds disfavor application in cardiovascular surgery due to poor biomechanics and rapid degradation. Herein, we proposed a green strategy to crosslink and functionalize acellular scaffolds via the self-assembly of copper@tea polyphenol nanoparticles (Cu@TP NPs), and the resultant nanocomposite acellular scaffolds were named as Cu@TP-dBPs. The crosslinking degree, biomechanics, denaturation temperature and resistance to enzymatic degradation of Cu@TP-dBPs were comparable to those of glutaraldehyde crosslinked decellularized bovine pericardias (Glut-dBPs). Furthermore, Cu@TP-dBPs were biocompatible and had abilities to inhibit bacterial growth and promote the formation of capillary-like networks. Subcutaneous implantation models demonstrated that Cu@TP-dBPs were free of calcification and allowed for host cell infiltration at Day 21. Cardiac patch graft models confirmed that Cu@TP-dBP patches showed improved ingrowth of functional blood vessels and remodeling of extracellular matrix at Day 60. These results suggested that Cu@TP-dBPs not only had comparable biomechanics and biostability to Glut-dBPs, but also had several advantages over Glut-dBPs in terms of anticalcification, remodeling and integration capabilities. Particularly, they were functional patches possessing antibacterial and proangiogenic activities. These material properties and biological functions made Cu@TP-dBPs a promising functional acellular patch for cardiovascular applications.

## Graphical Abstract

**Figure:**
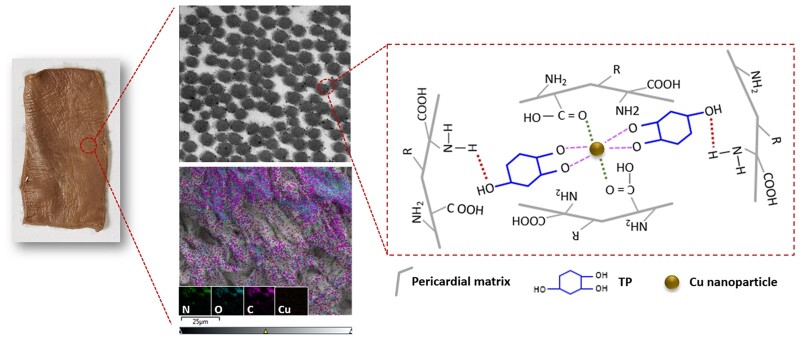

## Introduction

Cardiovascular disease, which represents a group of diseases affecting the heart and circulatory system, is a leading cause of morbidity and mortality throughout the world. Regardless of etiology, the application of patches is a widely accepted therapeutic method for surgical treatment of cardiovascular disease, including repair and reconstruction of myocardium, heart valves and blood vessels [1, 2]. Currently, patches commonly used in clinically consist of synthetic patches and xenogenic biological patches. The major drawback of synthetic patches such as woven nylon and expanded polytetrafluoroethylene is that they are rigid and induce reactive inflammation and thrombosis upon implantation [3]. Xenogenic biological patches offer several advantages over synthetic patches, which include good pliability, lower thromboembolism and decreased suture line bleeding [4]. Glutaraldehyde (Glut) is a conventional crosslinker to mask xenoantigenicity and improve the biomechanics and biostability of biological patches. However, Glut crosslinked patches are prone to calcification that may lead to long-term failure [5, 6]. Delayed remodeling and integration with surrounding tissue are also important limitations of these patches due to reconstructive failure [7]. Decellularization is an alternative method for processing biological tissues with decreased antigenicity, resistance to calcification and favorable tissue remodeling [8]. However, non-crosslinked acellular patches have low mechanical properties and exhibit rapid degradation rates both *in vitro* and *in vivo*, disabling their application in cardiovascular surgery [9, 10].

Hence, it is desirable to explore a new strategy to crosslink acellular patches suitable for cardiovascular applications. In addition, future cardiovascular patches should be functional materials to have desired biological activities.

Copper is an essential metal element to all organisms, as copper ion acts as a cofactor for many enzymes involved in redox reactions and various biological processes. Particularly, copper ions have been found to have proangiogenic and antibacterial activities [11, 12]. In this context, there has been a growing tendency to incorporate copper ions or copper-based nanoparticles (acting as a reservoir of copper ions) into biomaterials to fabricate functionalized biomaterials [13–15].

Green tea is a popular consumed beverage in Asia that has attracted more attention in recent years. Tea polyphenol (TP) is a general term of polyphenolic mixtures extracted from green tea, which has inherent antibacterial, antioxidant and antiradical activities [16, 17]. It also serves as a good reducer of metal ions, thus favoring the green synthesis of metal nanoparticles [18, 19]. Furthermore, it has been reported that polyphenols derived from plants can be utilized as natural crosslinking agents, as they can be self-assembly with protein side chains via covalent or non-convent bonds [20].

In this study, we used decellularized bovine pericardia (dBPs) as the patch scaffolds and proposed a green strategy to crosslink and functionalize acellular patches via the self-assembly of copper@tea polyphenol nanoparticles (Cu@TP NPs). We hypothesized that Cu@TP NPs were able to bridge and crosslink dBPs, thus improving the biomechanical properties and biostability of dBPs. Furthermore, the introduction of Cu@TP NPs could endow dBPs with biological functions, such as antibacterial and proangiogenic activities.

## Materials and methods

Detailed materials and methods are available in the Supplementary Materials.

### Fabrication of Cu@TP-dBPs

Fresh bovine pericardia were harvested from a local abattoir (Wufeng Company, China) and transported on ice to the laboratory. Decellularization was performed following our previously established method [21]. The obtained dBPs were sterilized using 70% alcohol and rinsed in sterile phosphate buffered saline (PBS). To prepare TP solution, the decaffeinated green tea (10 g) was added to 100 mL double distilled water and heated at 60°C for 60 min. Subsequently, dBPs were immersed in copper sulfate (CuSO_4_, Sigma Aldrich, Germany) solution for 48 h at 37°C. After washing with PBS, dBPs loaded with copper ions then were treated with TP solution for 12 h at 40°C. For comparison purposes, dBPs, dBPs treated with TP solution (TP-dBPs) and dBPs treated with Glut (Glut-dBPs) were prepared. Glut crosslinking was performed as the previous study [22]. TP-dBPs were prepared by incubating dBPs in TP solution for 12 h at 40°C.

### Physicochemical characterization of Cu@TP-dBPs

Physicochemical characterization of Cu@TP-dBPs were studied by transmission electron microscopy (TEM), scanning electron microscopy (SEM), energy dispersive X-ray spectroscopy (EDS) and Fourier transform infrared (FTIR) spectroscopy. Hydrodynamic size and zeta potential were measured by Zetasizer Nano-ZS90 [23, 24]. A ninhydrin assay was performed to measure the amount of free amino groups of each samples using a commercially available kit (GenM3d, USA). Degree of crosslinking is calculated following the equation:

Degree of crosslinking (%) = (1 – amine content in sample/amine content in non-crosslinked samaple) × 100%.

### Extract cytotoxicity assay

Extract cytotoxicity was determined by CCK-8 assay (Dojindo, China) using L929 cells. According to the International standard ISO 10993-5 regarding tests for *in vitro* cytotoxicity of medical devices, materials leading to a cell viability result above 70% of the control were considered as non-cytotoxic.

### Hemolysis assay

Hemolysis assay was performed using blood from donors. Samples (∼1 cm^2^) were placed in the sterile Eppendorf tube and 1 mL diluted red blood cell (RBC) solution was added per tube and incubated at 37°C for 3 h. The RBCs incubated in deionized water and PBS were used as the positive and negative controls, respectively. The hemolysis rates were calculated using the following equation:

$$\text{Hemolysis rate}\;\left(\%\right)=\left(OD_{\text{test}}-OD_{\text{neg}}\right)/\left(OD_{\text{pos}}-OD_{\text{neg}}\right)\times 100\%,$$

where *OD*_test_, *OD*_neg_ and *OD*_pos_ were the *OD*_545_ values of samples, negative control and positive control, respectively.

### Differential scanning calorimetry

Differential scanning calorimetry (DSC) was used to measure the thermal denaturation temperature (Td) of the tested pericardial samples using a DSC 2500 Differential Scanning Calorimeter (TA Instruments, USA). The resultant heating curves were analyzed using Thermal analysis software and the denaturation temperature was recorded at the height of the endothermic peak.

### *In vitro* collagenase assay

Samples were lyophilized, cut into pieces (∼ 1 mm^3^) and weighed (initial dry weight). Then, the samples (40 mg) were treated with collagenase Type I (1.5 mg/mL, Sigma Aldrich, Germany). At every predetermined time point, the samples were lyophilized and weighed again (final dry weight). The degree of enzymatic degradation of the samples was quantified as the percent weight loss (*W*%), which is calculated using the following formula:

$$W\%=\left(W_{0}-W_{t}\right)/W_{0}\times 100\%,$$

where *W*_0_ represents the initial weight of samples and *W*_t_ represents the weight of corresponding sample after enzymatic degradation treatment.

### Tensile testing

Experiments were carried out using a Zwick tensile tester (Zwick GmbH & Co. KG). Pericardial samples of the same directions were cut into 50 (length) × 10 (width) mm rectangular strips. The mean thickness of each sample was determined by a series of measurements at four different points using a Mitutoyo digital micrometer. Samples were attached to grips. The tensile testing was performed at 5 mm/minute until failure. All testing was conducted at room temperature.

### *In vitro* antibacterial activity assay

*Staphylococcus aureus* (*S. aureus*, Gram-positive bacteria) suspension was inoculated on nutrient agar plates. Samples were cut into round pieces with a diameter of 15 mm and placed on the surface of agar plates, and co-cultured with *S. aureus* for 12 h at 37°C. The clear area indicating zone of inhibition was measured and recorded.

### *In vitro* tube formation assay

The tested pericardial samples (∼ 0.5 mm^3^/well) were placed in six-well plates and incubated in endothelial medium (2 mL) at 37°C. The conditional medium from samples were collected at Day 3. Human umbilical vein endothelial cells (HUVECs) (3 × 10^4^ cells/well) were resuspended in conditional medium and seeded onto the solidified Matrigel-coated wells. After 12 h of incubation, cells were stained with phalloidin (Servicebio, China) and 4',6-diamidino-2-phenylindole (Sigma Aldrich, Germany) solution.

### Subcutaneous implantation models

The animal experiments were performed according to the NIH Guide for the Care and Use of Laboratory Animals, and all protocols were approved by the Institutional Animal Care and Use Committee of Changhai Hospital. To evaluate the *in vivo* responses including biostability, cell ingrowth and calcification, the tested samples were implanted subcutaneously into Sprague Dawley rats. At 21 days, rats were scarified and patch explants were harvested and then possessed for histological analysis and calcium quantitative analysis.

### Cardiac patch graft models

To evaluate the capacity of integration with myocardial tissues, the tested samples were implanted to the heart of rats. Briefly, rats were anesthetized and heart was exposed through a median sternotomy. The samples were cut into round patches (∼ 5 mm) and laid on the top of cardiac wall and sutured to the margin of the patch with around tissues. At 60 days, rats were scarified and hearts were harvested and then possessed for histological analysis.

### Histological and immunohistochemical evaluation

The specimens were fixed in 4% buffered formaldehyde for 24 h, processed into paraffin and then sectioned at 5 μm. Sections were deparaffinized and stained with hematoxylin and eosin (HE) for morphological examination, with Verhoeff’s Van Gieson (VG) staining for collagen and elastin, and with alizarin red S staining for detection of calcification. The expression of α-smooth muscle actin (α-SMA) was evaluated using immunohistochemistry.

### Calcium quantitative analysis

Explants were weighed and decalcified in 6 N HCl at room temperature for 3 days. The calcium content of the 0.6 N HCl supernatant was determined colorimetrically using alizarin red S as indicator. The total calcium content of each explants was normalized to its dry weight.

### Statistical analysis

Results are expressed as mean ± standard error (SD). Statistical analyses were performed using GraphPad Prism 6.0 (GraphPad Software, USA). For normal distributions, the differences between two of the groups were evaluated by means of the t-test, while one-way analysis of variance for multiple samples. When the data distribution was not normally distributed, Mann–Whitney or Kruskal–Wallis non-parametric multiple-comparison test was employed. *P *<* *0.05 was considered statistically significant.

## Results

### Fabrication of Cu@TP-dBPs

**Figure 1.**
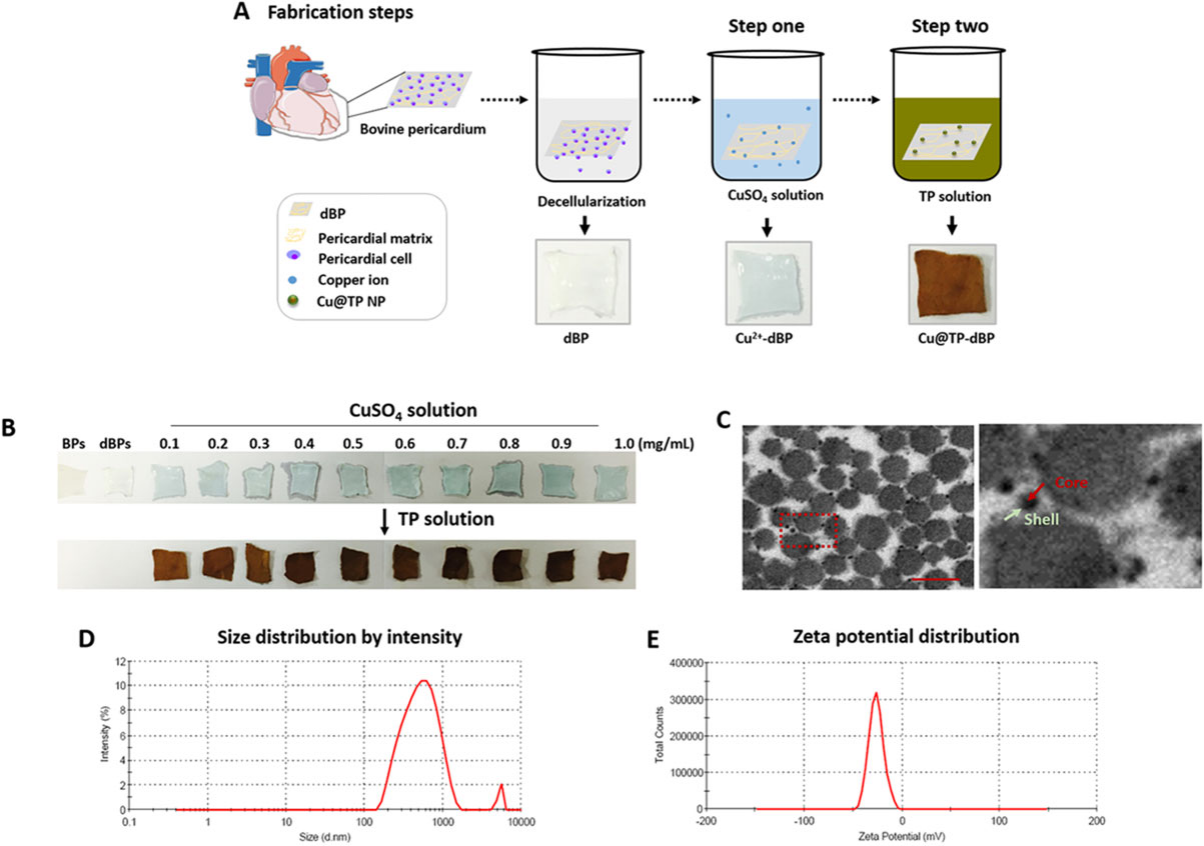
*In situ* synthesis of Cu@TP NPs within dBPs. Schematic diagram (**A**) and gross images (**B**) of Cu@TP-dBP fabrication. Representative images of TEM (**C**) of Cu@TP-dBPs. The hydrodynamic size (**D**) and the zeta potential (**E**) of Cu@TP NPs in buffer solution. Scale bar = 100 nm

Cu@TP-dBPs were crosslinked using a two-step method (Fig. 1A). First, dBPs were immersed in the CuSO_4_ solution to load copper ions (Cu^2+^-dBPs). As shown in Fig. 1B, an obvious color change was observed in CuSO_4_ solution-treated dBPs from white to blue. Subsequently, dBPs loaded with copper ions were treated with TP solution (polyphenolic content, 2.89 mg/mL). The resultant Cu@TP-dBPs showed a yellow brown color, and the color gradually became darker with the concentration of copper ions rising higher. TEM images (Fig. 1C) showed that Cu@TP NPs were synthesized at the meantime, which were approximately spherical in shape with nanoscale size, and compactly bound to matrix fibers of dBPs. From the TEM image, we found that the synthesized Cu@TP NPs showed a core@shell structure with deep-contrast core and light-contrast layer. We also examined the hydrodynamic size and zeta potential Cu@TP NPs in solution. The hydrodynamic size of Cu@TP NPs in solution (545.2 ± 106.2, Fig. 1D) was much larger than that acquired by TEM, which may because the porous structure of dBPs limited the growth of Cu@TP NPs. The zeta potential of Cu@TP NPs were –25.2 ± 3.1 mV (Fig. 1E). This negative zeta potential was due to the capping of TPs, which were possessing negative charge.

### Physicochemical characterization of Cu@TP-dBPs

**Figure 2.**
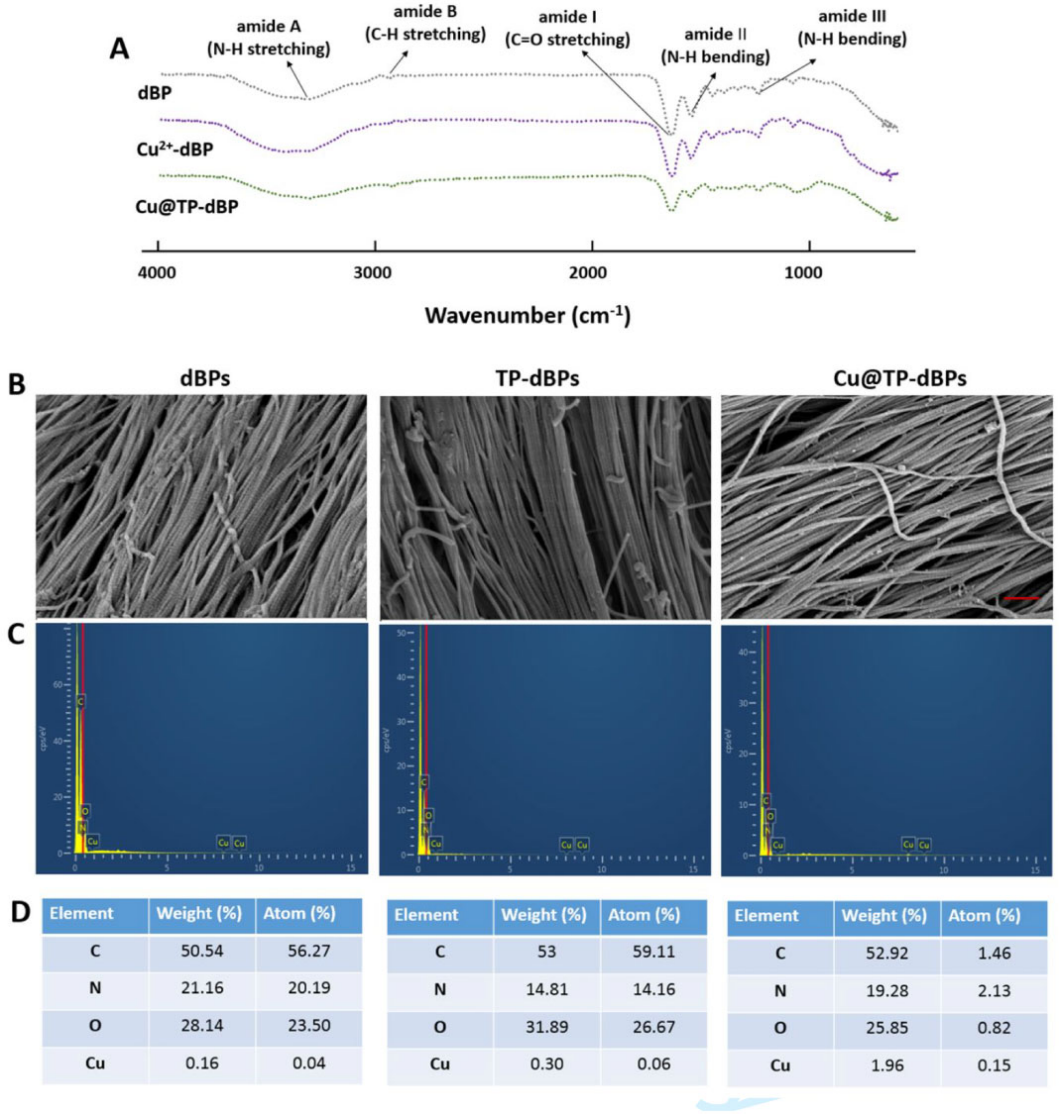
FTIR and SEM-EDS analysis of Cu@TP-dBPs. FTIR spectra of dBPs, Cu^2+^-dBPs, Cu@TP-dBPs (**A**). SEM top-view of surface microstructures (**B**), EDS spectra of corresponding SEM images (**C**) and elemental compositions in the interior detected by EDS maps (**D**): C, carbon; N, nitrogen; O, oxygen; Cu, copper. Scale bar = 600 nm

The self-assembly of Cu@TP NPs with dBPs was evaluated by FTIR spectroscopy. Compared to the FTIR spectrum of dBPs, the absorption peak of amide I band in dBPs loaded with copper ions from 1642.93 to 1639.92 cm^–1^ (Fig. 2A), suggesting the electrostatic interaction between copper ions and C = O groups of collagens [25]. Phenolic hydroxyl groups have a strong potential for binding and reacting with proteins by forming hydrogen bonds. Our results showed that a major shift to the lower frequency was observed at the band of amide A from 3325.80 to 3309.13 cm^–1^, suggesting the formation of hydrogen bonds between hydroxyl groups of TPs and NH groups of collagens. As a result, interactions of multiple molecules (copper–collagen, TP–collagen and copper–TP) bridged and crosslinked dBP collagens.

The surface morphology of Cu@TP-dBPs was examined by SEM. As shown in Fig. 2B, the presence of Cu@TP NPs was clearly visible on the surface of Cu@TP-dBPs, and they strongly adhered to the collagen. We also compared the collagen structural differences between Cu@TP-dBPs, dBPs and TP-dBPs. Collagen alignment was not different among the three groups, suggesting the synthesized nanoparticles did not alter the collagen structure of dBPs. The EDS mappings of the SEM images revealed enhanced copper signals in the Cu@TP-dBPs (Fig. 2C and D), suggesting successful copper incorporation.

### Biocompatibility of Cu@TP-dBPs

**Figure 3.**
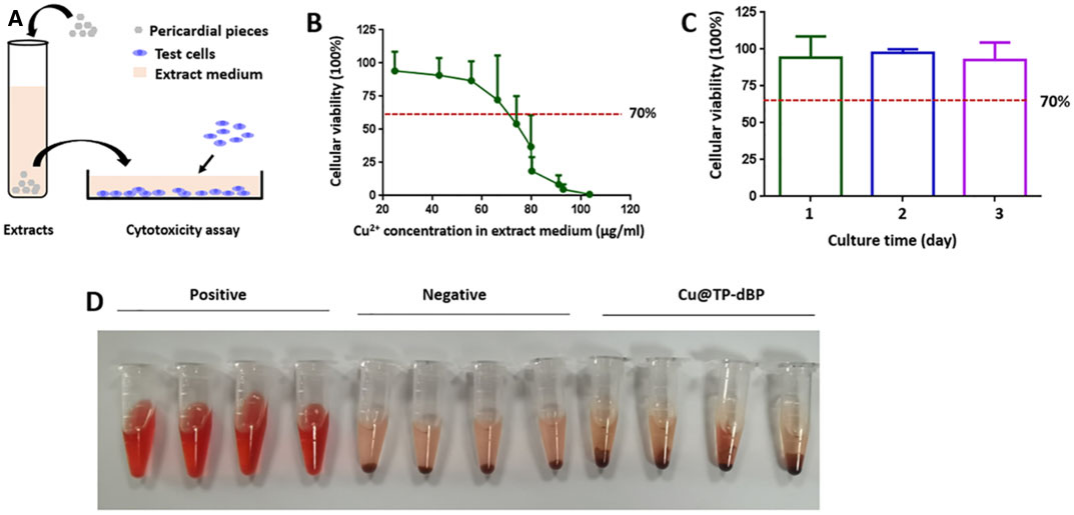
Cytocompatibility and blood biocompatibility of Cu@TP-dBPs. Schematic illustration showing the experimental design for extract toxicity assay (**A**). The tested cells were treated with extract medium for 1 day (**B**) or 3 days (**C**) and cellular viability of L292 cells was determined by CCK-8 assay. Hemolysis assay (**D**) of Cu@TP-dBPs fabricated using 0.1 mg/mL CuSO_4_ solution had no hemolytic activity. *n* = 4

Non-toxicity is a prerequisite for the application of biomaterials in clinical application. Therefore, we next assessed the appropriate copper ion loading concentration without toxic effects on mammalian cells using the extract toxicity assay. Cu@TP-dBPs were fabricated using CuSO_4_ solution at concentrations of 0.1–1 mg/mL. They were minced into pieces and incubated in culture medium to prepare extract medium (Fig. 3A). The corresponding copper ion concentration in extract medium is shown in Supplementary Table S1. CCK-8 results revealed that the cellular viability of L929 cells decreased gradually with increasing copper ion concentration (Fig. 3B). According to the International safety standard of 70% viability (ISO 10993-5), there was no significant toxicity on L929 cells when Cu@TP-dBPs were fabricated using 0.1 mg/mL CuSO_4_ solution even at Day 3 (Fig. 3C). Although Cu@TP-dBPs fabricated with 0.2, 0.3 and 0.4 mg/mL CuSO_4_ solution were safe for L929 cells at Day 1, cell viability significantly decreased when cells were cultured for the other two days (Supplementary Fig. S1). Accordingly, Cu@TP-dBPs were fabricated using 0.1 mg/mL CuSO_4_ solution in the following experiments as it was the highest non-toxic concentration.

Having determined that the Cu@TP-dBPs fabricated using 0.1 mg/mL CuSO_4_ solution had a favorable cytocompatibility, we further assessed their suitability for blood biocompatibility via a RBC hemolysis assay *in vitro*. Purified water and PBS showed positive (hemolysis) and negative (non-hemolysis) phenomena (Fig. 3D), respectively. The hemolysis ratio of Cu@TP-dBPs were 1.87 ± 1.28%, far below the International safety standard of 5% (ISO10993-4:2002), suggesting that the fabricated Cu@TP-dBPs could be used for blood-contacting applications.

### Crosslinking properties of Cu@TP-dBPs

**Figure 4.**
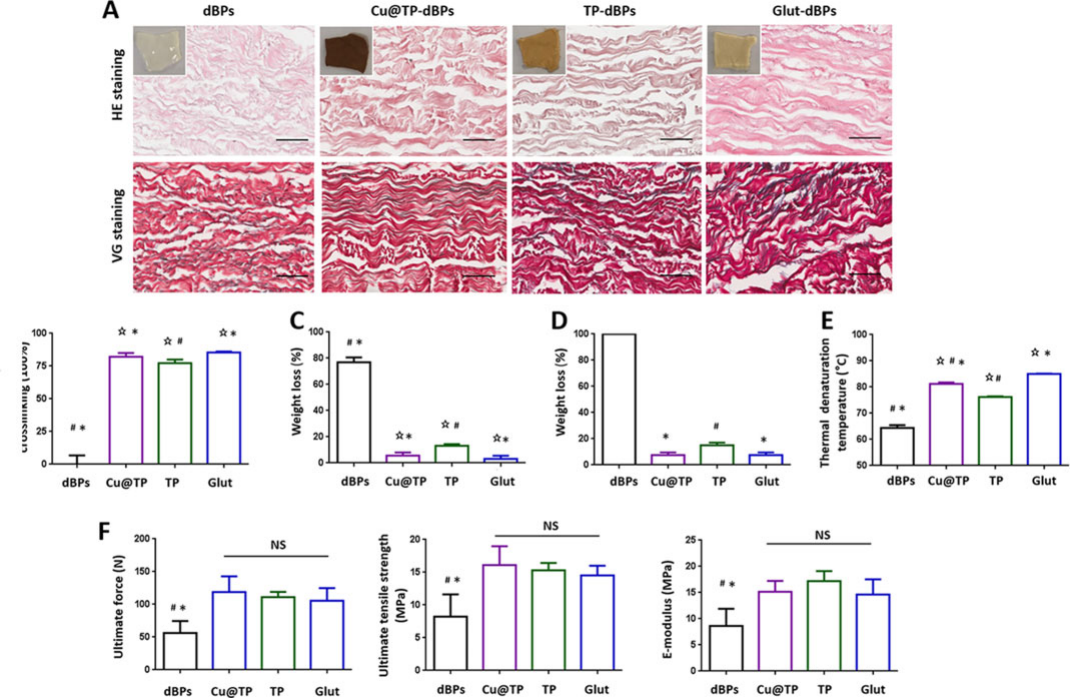
Crosslinking characterizations of Cu@TP-dBPs. Representative histological staining of the four groups (**A**). Small image showed gross images of dBPs. Ninhydrin assay (**B**), collagenase assay at Day 3 (**C**) and at Day 14 (**D**), DSC (**E**) and uniaxial tensile testing (**F**). ⋆*P* < 0.05 compared to dBPs, # compared to Glut-dBPs, * compared to TP-dBPs, NS, not significant. Scale bar = 50 µm

Histological staining showed that all pericardial samples were free of cells with well-preserved extracellular matrix after decellularization (Fig. 4A). Ninhydrin assay was performed to determine the degree of crosslinking. Using dBPs as the standard of 100% amine groups, the crosslinking degree increased in the order TP-dBPs < Cu@TP-dBPs < Glut-dBPs (Fig. 4B). The crosslinking degree of TP-dBPs was lower than that of Cu@TP-dBPs (*P *=* *0.0286), suggesting that the synthesized Cu@TP NPs might enhanced the crosslinking effect of TPs through copper–collagen and copper–TP interactions. No significant difference was found between Cu@TP-dBPs and Glut-dBPs (*P *=* *0.0571).dBPs were very susceptible to Type I collagenase, they had >70% weight loss at Day 3 (Fig. 4C), and completely degraded at Day 14 (Fig. 4D). Crosslinking was able to prevent rapid degradation of dBPs, and the three crosslinked dBPs (TP-dBPs, Cu@TP-dBPs and Glut-dBPs) had <20% of weight loss at Day 14. The weight loss of TP-dBPs was higher than that of Cu@TP-dBPs (*P *=* *0.0020) and Glut-dBPs (*P *=* *0.0020) at Day 14. However, no significant difference was found between Cu@TP-dBPs and Glut-dBPs (*P *=* *1.0000) as for collagen stability against Type I collagenase.

Having determine the enzymatic stability of Cu@TP-dBPs, we next examined the collagen thermal stability assessed by DSC. The collagen denaturation temperature increased in the order dBPs < TP-dBPs < Cu@TP-dBPs < Glut-dBPs (Fig. 4E and Supplementary Fig. S2). Particularly, the collagen denaturation temperature of Cu@TP-dBPs was only secondary to that of Glut-dBPs.

Crosslinking is also used to strengthen biomechanical properties of biomaterials. Tensile testing (Fig. 4F) showed that no significant differences in ultimate force (*P *=* *0.6169), ultimate tensile strength (*P *=* *0.5555) and elastic modulus (*P *=* *0.3084) were found among Cu@TP-dBPs, TP-dBPs and Glut-dBPs. Taken together, these results suggested that Cu@TP-dBPs showed great biomechanics and collagen stability comparable to those of Glut-dBPs.

### Antibacterial and proangiogenic activities of Cu@TP-dBPs

**Figure 5.**
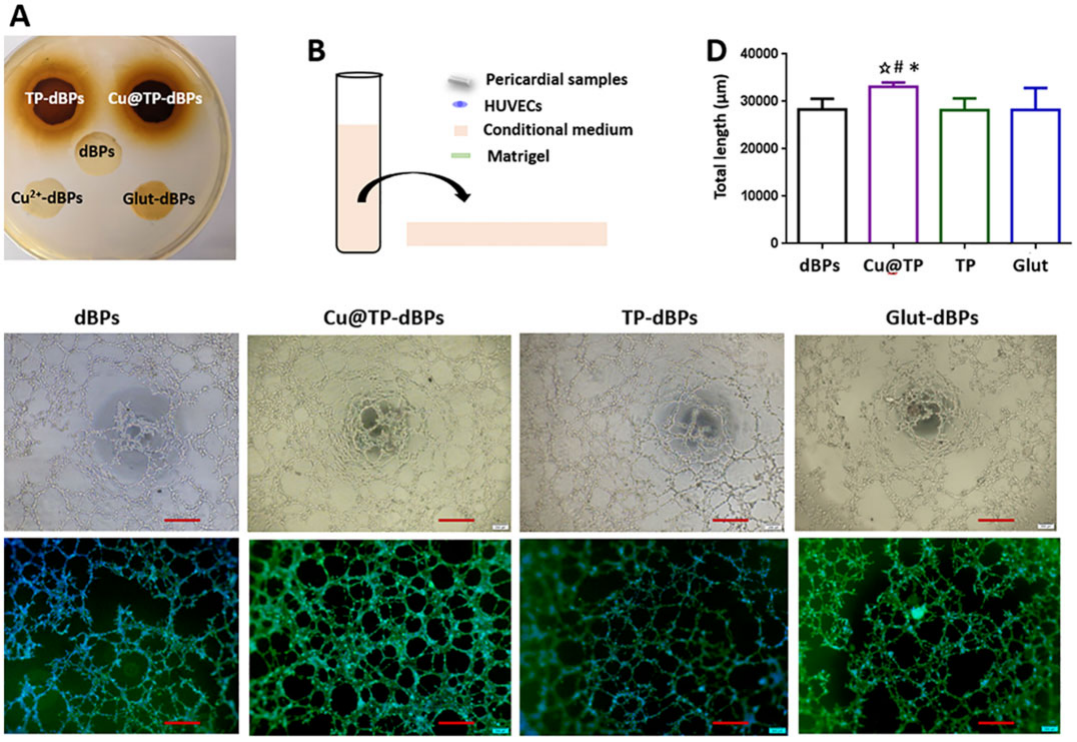
Antibacterial and proangiogenic activities of Cu@TP-dBPs. Inhibition zone assay of the tested patches (**A**). Schematic illustration showing the experimental design for angiogenesis assay (**B**). Representative phase contrast microphotographs showing the formation of capillary-like networks (**C**). Upper: bright images. Lower: immunofluorescence images of phalloidin and DAPI double-staining. Quantitative analysis of specific parameters of capillary tube formation after 12-h incubation on Matrigel (**D**). *P < 0.05 compared to dBPs, # compared to Glut-dBPs, ⋆ compared to TP-dBPs. Scale bar = 400 µm. All *n* = 4

*Staphylococcus* *aureus* is a leading cause of endocarditis and cardiovascular surgical site infections [26]. Therefore, we used *S. aureus* to evaluate the antibacterial activity of Cu@TP-dBPs. As shown in Fig. 5A, clear inhibition zones were observed around TP-dBPs (1.90 [1.83–1.98] mm) and Cu@TP-dBPs (1.90 [1.83–1.90] mm), and no significance was recognized statistically between them (*P *>* *0.9999), suggesting that both of them had antibacterial activity against *S. aureus*.

To investigate the effects of TP-dBPs, Cu@TP-dBPs and Glut-dBPs in angiogenesis, we employed an *in vitro* tube formation assay. As shown in Fig. 5B, HUVECs were resuspended in pericardial conditional medium and seeded onto the solidified Matrigel-coated wells. HUVECs cultured in Cu@TP-dBP conditional medium showed an enhanced ability to form capillary-like structures (Fig. 5B) with increased tube length compared to dBPs (Fig. 5C, *P *=* *0.0209). In contrast, HUVECs cultured in TP-dBPs (*P *=* *0.9497) or Glut-dBPs (*P *=* *0.9771) showed a reduced ability to form capillary-like structures when compared with dBPs although no statistical significance was reached (Fig. 5D). Taken together, these results suggested that Cu@TP-dBPs were functional biomaterials with antibacterial and proangiogenic activities.

### Cu@TP-dBPs were free of calcification in a rat subcutaneous implantation model

**Figure 6.**
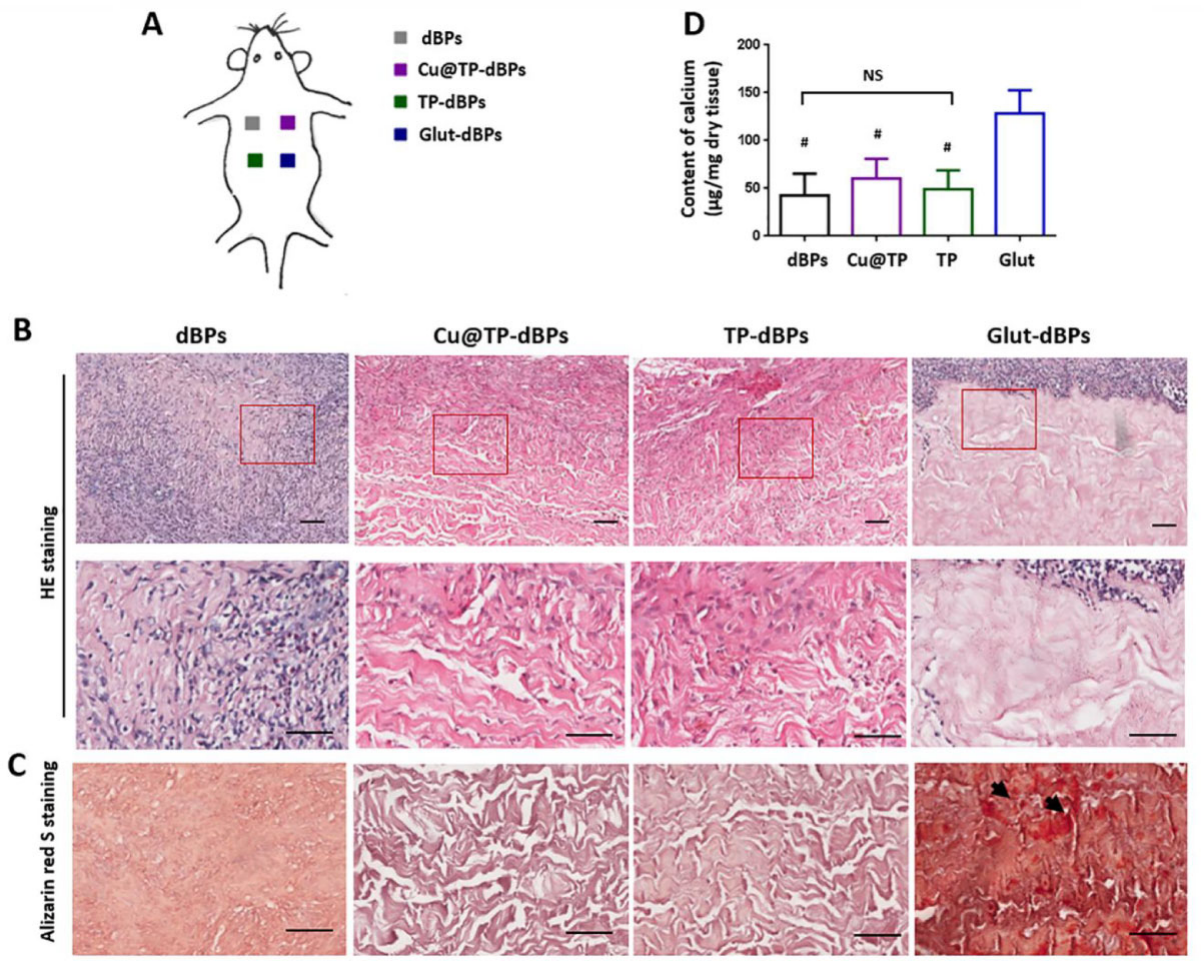
Recellularization and calcification of Cu@TP-dBPs in the rat subcutaneous implantation models. Schematic illustration showing the experimental design for rat subcutaneous implantation models (**A**). HE staining (**B**), alizarin red S staining (**C**) and quantification of calcium content (**D**) of the patch explants. Lower panel showed the higher magnification images of corresponding samples at the regions marked by red-boxes. ^#^*P* < 0.05 compared to Glut-dBP explants. NS, not significant. Arrow: calcium deposition. Scale bar = 50 µm. *n* = 4

To evaluate the *in vivo* host response, the tested pericardial samples were implanted subcutaneously in rats for 21 days (Fig. 6A). HE staining showed that dBPs were almost degraded and completely infiltrated by host cells (Fig. 6B). The surrounding acellular regions of both Cu@TP-dBP explants and TP-dBP explants were infiltrated by a number of fibroblast-like cells, whereas the cells were hard to infiltrate into Glut-dBP explants. In addition, Glut-dBP explants showed focal calcium deposition, while no visible calcification was found in explants of the other three groups (Fig. 6C), confirming that Glut crosslinking accelerated the calcification process. The differences of calcium content among the four groups were further characterized in terms with quantitative analysis (Fig. 6D). Consistent with the histological staining, the highest calcium content was found in Glut-dBP explants. No significant difference in calcium content was found among dBP, TP-dBP and Cu@TP-dBP explants (*P *=* *0.5353), suggesting that Cu@TP-dBPs showed anticalcification potential.

### Cu@TP-dBPs showed good handling properties and myocardial integration in a rat cardiac patch graft model

**Figure 7.**
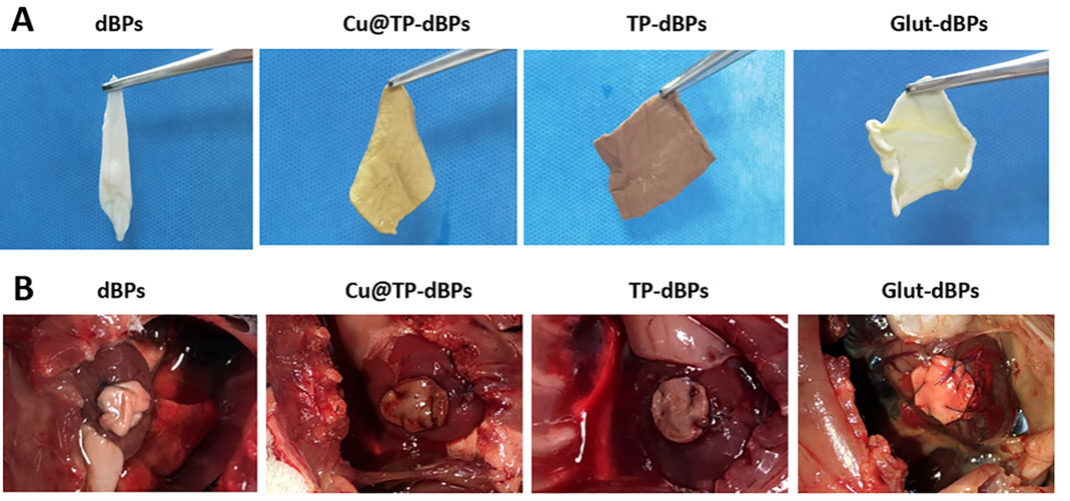
Ease of handling and *in vivo* toxicity of Cu@TP-dBPs. After crosslinking, dBPs became stiffer (**A**). Cu@TP-dBPs, TP-dBPs and Glut-dBPs could be easily trimmed to the desired shape and size and sutured with myocardium (**B**)

Having determined that Cu@TP-dBPs were more permissive to recellularization than Glut-dBPs in the rat subcutaneous implantation model, we next investigated their remodeling and integration capacities in a rat cardiac patch graft model. In the perspective of surgical handing, dBPs were too soft to handle, trim and suture, thus, they were inconvenient for surgical procedures. In contrast, the stiffener Cu@TP-dBPs, TP-dBPs and Glut-dBPs had better handling properties and could be easily trimmed to the desired shape and size and sutured with myocardium (Fig. 7A and B).

**Figure 8.**
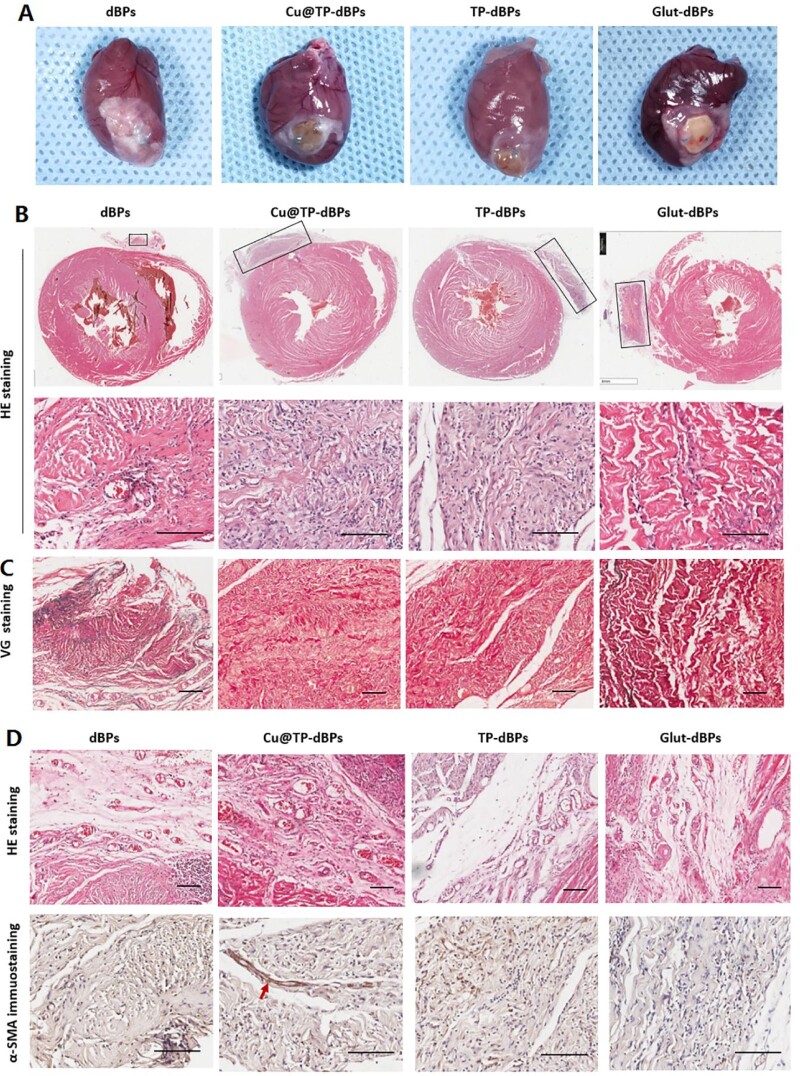
Myocardial integration of Cu@TP-dBP patches in the rat cardiac patch graft models. Gross examination showing that Cu@TP-dBP patches integrated well with surrounding myocardium (**A**). HE staining (**B**) and VG staining (**C**) showing that Cu@TP-dBP patches degraded faster and displayed improved recellularization than Glut-dBP patches *in vivo*. HE staining and α-SMA immunohistochemical staining showed that Cu@TP-dBP patches displayed enhanced blood vessel formation (**D**). Scale bar = 100 µm. *n* = 3

After implantation for 60 days, the hearts were harvested and assessed for histological features. Grossly, all patches were surrounded by thin fibrous capsules (Fig. 8A). dBP patches were almost degraded, while Glut-dBP patches still preserved their primary structures with a foreign mass protruding from the surface of the heart (Fig. 8B, upper panel). Cu@TP-dBP and TP-dBP patches showed favorable recellularization (Fig. 8B, lower panel), which is a crucial step for integration of implanted patches with native tissues. VG staining (Fig. 8C) revealed that neomatrix development within the Cu@TP-dBP patches, suggesting the remodeling of extracellular matrix. However, Glut-dBP patches showed limited remodeling with poor recellularization.

As shown in Fig. 8D, the spaces between Cu@TP-dBP and surrounding myocardium contained a matrix richer in blood vessels than the loose connective tissue seen surrounding the other patches (Fig. 8D, upper panel). Functional blood vessels with medial layers were determined by the immunohistochemical staining for α-SMA-positive medial layers. They were found to penetrate into Cu@TP-dBP patches (Fig. 8D, lower panel, as indicated by red arrow), while the depth areas of Glut-dBP and TP-dBP patches lacked of functional blood vessels. Functional blood vessels were hardly found in dBP patches, which mainly due to the rapid degradation of these patches. Taken together, these results suggested that Cu@TP-dBP patches had controlled degradation, and showed favorable recellularization, ingrowth of functional blood vessels and remodeling of extracellular matrix, which could promote tissue integration with surrounding myocardial tissues.

We evaluated calcification in the rat cardiac patch graft model using alizarin red S staining. No calcification was noted in the dBPs, Cu@TP-dBPs and TP-dBPs; however, local calcification was found in the Glut-dBPs (Supplementary Fig. S3). In addition, we investigated the *in vivo* toxicity of Cu@TP-dBPs on the major organs, which were collected during *in vivo* animal experiments. As shown in Supplementary Fig. S4, no necrosis, inflammation, hemorrhage or other obvious damage was found in brain, lung, kidney, liver and spleen, confirming that the fabricated Cu@TP-dBPs exhibited negligible *in vivo* toxicity.

## Discussion

Patch repairing and replacement are common especially in the cardiovascular diseases. Generally, two main types of patch scaffold have been proposed and developed: (i) artificial patches, fabricated from synthetic and natural (biological) polymers; and (ii) native biological patches from allogeneic or xenogenic sources [27, 28]. Patches fabricated from synthetic polymers such as expanded polytetrafluoroethylene and polyethylene terephthalate exhibit enough mechanical properties and controlled biodegradability but bad biocompatibility, which cause high risk of postoperative complication. The patches fabricated from natural polymers such as collagen and hyaluronic acid show good biocompatibility but bad mechanical strength. Xenogenic tissues such as porcine or bovine pericardium is the main sources of native biological patches because human tissue are in short supply.

In order to minimize the immunogenic components, xenogenic tissues need to be treated with crosslinking agents or underwent decellularization. Currently, decellularized patches show promising applications in tissue engineering, and several types of commercial decellularized patches (i.e. Veritas Collagen Matrix^®^, Peri-Guard^®^ and Tutopatch^®^) have been already approved for clinical use [29]. However, patches for cardiovascular applications should have enough mechanical properties and structural stability [29]. For example, it is necessary for cardiac patches to hold the ventricular pressure, withstand the tensile force generated by wall contraction and provide mechanical support to prevent cardiac dilation [30, 31]. Patches used for leaflet repair in atrioventricular valves or as a leaflet need to tolerate repeating cycles of mechanical load for years. Native biomaterials often exhibit poor mechanical properties and rapid enzyme degradation. Upon implantation, uncrosslinked natural biomaterials are subject to chemical and enzymatic degradation, seriously affecting the mechanical properties and decreasing the life of the patches. Pavcnik *et al.* [32] reported a high failure rate of CorMatrix^®^ at 3–4 months in an ovine model of carotid artery grafting due to dilatation, stenosis, dissections and aneurysm formation. Furthermore, accumulating evidence have shown that decellularized patch CorMatrix^®^ have good performance in the low pressure, usually extracardiac environment (i.e. veins), but when they were used at higher pressure intracardiac sites such as the aortic valve or in semilunar valve, complications are more likely to occur [33]. Hence, it is desirable to explore a new strategy to crosslink acellular patches to improve their mechanical strength and biological stability.

Plant polyphenols have been used to crosslink biological tissues by the formations of non-convent bonding between matrix proteins and polyphenols [34, 35]. Polyphenols are also able to coordinate with transition metal ions by forming stable chelating rings and reduced them to atoms, and in the meantime, hydroxyls are oxidized to corresponding quinones and attached onto the metal surface to form a core@shell structure [36]. In addition, transition metal coordination has been emerging as an important class of supramolecular crosslinkers that can enhance the mechanical properties of collagen materials [37]. Based on the above mechanisms, we speculated that copper, collagen and TP were able to form multiple molecular interactions within the self-assembled structure. Consistent with our hypothesis, we found that copper ions interacted with dBP collagens through electrostatic interaction. Subsequently, TPs were coordinated with the entrapped copper ions to form Cu@TP NPs with a core@shell structure. Meanwhile, the assembly of TPs was able to interact with dBP collagens by the formation of hydrogen bonding. It was noted that these multiple molecular interactions (copper–collagen, TP–collagen and copper–TP) could achieve a higher crosslinking degree than the simple interaction between TPs and dBP collagens.

Glut is a widely used crosslinker for biological tissues, due to its high efficiency of collagen-based materials. However, calcification is a common and important problem for Glut crosslinked biomaterials. One of the mechanism is that Glut accelerates the calcification process by introducing the binding sites to the calcium ion [38, 39]. In this study, we found that Cu@TP-dBPs were free of calcification, suggesting that Cu@TP-dBPs showed anticalcification potential. Furthermore, our results showed that physical parameters of Cu@TP-dBPs, such as thermal denaturation, biodegradation and biomechanics, were comparable to those of Glut-dBPs. These results suggested that Cu@TP-dBPs were reliable biomaterials and suited for cardiovascular applications. This was a “green” strategy to crosslinking acellular patches, given that the process is gentle, and environmentally friendly without use of harsh, toxic and expensive chemicals.

Recellularization is the initial and crucial step for tissue constructive remodeling of acellular biomaterials *in vivo* [40, 41]. Rapid degradation has been recognized as an effective strategy to promote recellularization and constructive remodeling of biomaterials [42]. However, rapid degradation of cardiovascular patches may result in loss of mechanical strength to withstand the force of heart and vessels, which may ultimately contribute significantly to failure. Previous studies have demonstrated that plant polyphenols crosslinked biological tissues are initially resistant to collagenase and then show progressive degradation and host cell infiltration due to reversible non-covalent interactions and good biocompatibility [43, 44]. Consistently, we found that Cu@TP-dBPs in a rat cardiac patch graft model showed improved tissue integration capacity, including recellularization, ingrowth of functional blood vessels and remodeling of extracellular matrix. These results suggest that polyphenols based crosslinking strategy provided a control degradation process, which were favorable for patch integration with surrounding native tissues.

The combination of metal nanoparticles with synthetic or biological (i.e. collagen hydrogels and acellular tissues) materials provides an attractive approach to develop functional biomaterials [37]. Some studies used directly immersed or mechanical transfer methods to load mental nanoparticles in or on biological tissues. For example, Agarwal *et al.* [45] developed a mechanical transfer method using polyelectrolyte multilayers as transferred films to deliver silver nanoparticles onto biomedically relevant soft materials. Recently, some studies have showed that *in situ* synthesis of nanoparticles within material matrix is an effective approach to achieve functional modification of materials [37]. At the same time, the problem of nanoparticle aggregation could be avoided, as the free space within the hydrogel porous structure offers a nanoscopic pot for the synthesis of nanoparticles [46]. Therefore, we *in situ* synthesized Cu@TP NPs in this study.

Copper is a well-known antibacterial agent exhibiting bactericidal or bacteriostatic activity. For copper nanoparticles, the antibacterial activity is related to direct contact toxicity or release of dissolved copper ions from nanoparticles [12, 47]. In this study, we found that Cu@TP-dBPs had comparable antibacterial activity as TP-dBPs, suggesting that TP itself had enough antibacterial effect to inhibit bacterial growth. Indeed, TP as well as its polyphenol components have been found to inhibit growth, adherence, biofilm formation and protease activity of multiple bacteria, and thereby exhibiting broad antibacterial spectrum [48, 49].

Angiogenesis plays a crucial role in efficient wound healing and tissue regeneration, especially in cardiovascular patch implanting position. Copper has previously been suggested to be proangiogenic by multiple mechanisms that include releasing various angiogenic factors and stimulating endothelial cell proliferation [50, 51]. In this study, we found that Cu@TP-dBPs had an improved proangiogenic effect both *in vitro* and *in vivo*. *In vitro*, Cu@TP-dBP prompted HUVECs to form capillary-like structures. *In vivo*, Cu@TP-dBP patches showed favorable recellularization and formed functional blood vessels within the patches.

Although copper has many merits, the biocompatibility of copper ions is still debated, particularly in the case of copper nanoparticles. Previous studies reported that copper nanoparticles had potent cytotoxic effects on mammalian cells [52, 53]. In contrast, Shrikant Harne *et al.* [54] found that copper nanoparticles synthesized by green method hold excellent biocompatibility to HeLa, A549 and BHK21 cell lines. These discrepant results may be due to several factors, including the amount of released copper ions, the size and shape of the nanoparticles, and their capping chemistry [55]. To enhance biocompatibility of Cu@TP-dBPs, we assessed the appropriate copper ion loading concentration according to the results of extract toxicity assay. *In vitro* and *in vivo* studies confirmed that Cu@TP-dBPs fabricated using 0.1 mg/mL CuSO4 solution had good biocompatibility.

## Conclusions

In this study, we reported a strategy to crosslink and functionalize acellular patches on the basis of the self-assembly of Cu@TP NPs. Multiple molecular interactions (copper–collagen, TP–collagen and copper–TP) could bridge dBPs, and thereby improved the crosslinking effect of TPs. The resultant Cu@TP-dBPs showed comparable biomechanics and biological stability to Glut-dBPs, while they had several advantages over Glut-dBPs in terms of anticalcification, remodeling and integration capabilities. Furthermore, Cu@TP-dBPs were biocompatible and had antibacterial and proangiogenic activities. Collectively, these material properties and biological functions made Cu@TP-dBPs a promising functional acellular patch for cardiovascular applications.

## Supplementary data

Supplementary data are available at *REGBIO* online.

## Funding

This study was supported by grant from the National Key Research and Development Program of China (grant number 2016YFC1100900), National Natural Science Foundation of China (grant numbers 81770390, 82070402 and 82170376) and Key Research and Development Program of Ningbo (grant number 2018B10092).

*Conflict of interest statement*. None declared.

## Supplementary Material

rbac030_Supplementary_Data
