# Supplementary material for: Crosslinking and functionalization of acellular patches via the self-assembly of copper@tea polyphenol nanoparticles
Source: Regen Biomater. 2022 May 18;9:rbac030. doi: 10.1093/rb/rbac030 (PMC9157057; doi:10.1093/rb/rbac030)
Supplement: rbac030_Supplementary_Data [file rbac030_supplementary_data.doc]

**Supplementary data**

**Crosslinking and functionalization of acellular patches via the self-assembly of copper@tea polyphenol nanoparticles**

**Supplementary materials and methods**

**Decellularization of bovine pericardia**

Fresh bovine pericardia were harvested from a local abattoir (Wufeng Company, China) and transported on ice to the laboratory. Decellularization was performed following our previously established method [1]. Briefly, bovine pericardia immersed in 1% Triton X-100 (Sigma Aldrich, Germany) solution were frozen in a vacuum freeze drier and thawed at 37 °C and then processed with 1% Triton X-100 solution for another 48 hours. The obtained decellularized bovine pericardia (dBPs) were sterilized using 70% alcohol and rinsed in sterile phosphate buffered saline (PBS).

**Preparation of tea polyphenol (TP) solution**

To remove as much caffeine as possible and to inactivate the oxidizing enzymes, the dried green tea leaves (Xihu longjing, China) were blanched in water for 4 minutes at the optimal temperature of 100 °C and at a water-to-tea ratio of 20:1 mL/g, as reported in a previous study [2]. Subsequently, the blanched tea leaves were dried to constant weight at 70 °C. TP solution was prepared as previously reported, with some modifications [3]. Briefly, **t**he decaffeinated green tea (10 g) was added to 100 mL double distilled water and heated at 60 °C for 60 minutes. After cooling to room temperature, the obtained solution was centrifuged at 7000 revolutions per minute (rpm) and filtered. The TP content was measured using the Folin-Ciocalteu assay (Huich, China). The optical absorbance of the solutions was recorded with a spectrophotometer (EPOCH, USA) at 765 nm using gallic acid at various known concentrations as standard. The filtrated solution was kept in the refrigerator for further use.

**Fabrication of Cu@TP-dBPs**

dBPs were treated with copper sulfate (CuSO_4_, Sigma Aldrich, Germany) solution with a range of concentrations, and placed on a constant temperature shaker at 37 °C, 60 revolutions per minute (rpm) for 48 hours. Next, dBPs loaded with copper ions (Cu^2+^-dBPs) were washed with PBS to remove unbounding copper ions. Then, Cu^2+^-dBPs were treated with TP solution for 12 hours at 40 °C.

For comparison purposes, dBPs, dBPs treated with TP solutuion (TP-dBPs) and dBPs treated with [glutaraldehyde](https://www.sciencedirect.com/topics/medicine-and-dentistry/glutaraldehyde) (Glut-dBPs) were prepared. [Glut](https://www.sciencedirect.com/topics/medicine-and-dentistry/glutaraldehyde) crosslinking was performed as the previous study [4]. The dBPs were fixed in 0.6% Glut (Sigma Aldrich, Germany) in 50 mM 4-(2-hydroxyethyl)-1-piperazineethanesulfonic acid (HEPES)-buffered saline (pH 7.4) for 24 hours at room temperature. Subsequently, the solution was replaced by 0.2% Glut and stored for 6 days before used. TP-dBPs were prepared by incubating dBPs in TP solution for 12 hours at 40 °C.

**Transmission electron microscopy (TEM)**

Pericardial samples were dissected into 1 mm^3^ cubes and fixed in 2.5% (v/v) [Glut](https://www.sciencedirect.com/topics/medicine-and-dentistry/glutaraldehyde) for 2 hours at 4 °C. Subsequently, samples were washed with precooling PBS and dehydrated through a graded ethylalcohol series. Finally, the samples embedded in EPON resin that was polymerized overnight at 65 °C. Sections were prepared and stained with [uranyl acetate](https://www.sciencedirect.com/topics/medicine-and-dentistry/uranyl-acetate) and lead citrate. The microstructures were observed using a JEOL JEM-2100 transmission [electron microscope](https://www.sciencedirect.com/topics/medicine-and-dentistry/electron-microscope) (JROL, Japan).

**Scanning electron microscopy (SEM) and corresponding energy dispersive X-ray spectroscopy (EDS)**

To study the surface morphology of pericardial samples and their chemical composition, a Zeiss Ultra 55 scanning electron microscope (SEM, Zeiss, Germany) equipped with an energy dispersive X-ray spectrometer (EDS) was used. Images of samples were recorded using an operating voltage of 20 kV.

**Fourier transform infrared (FTIR) spectroscopy**

Pericardial samples were frozen and lyophilized and the acquisition of infrared spectra of all samples were carried out using a   FTIR Nicolet 6700 Transmittance spectrometer (Thermo Fisher, USA). Absorbance spectrums were recorded at a resolution of 8 cm^-1^ in the range of 4000 to 600 cm^-1^. Data were analyzed by quantitative peak information.

**Hydrodynamic size and zeta potential assays**

CuSO_4_ and TP solutions were mixed at a volume of at 1:1 ratio and then placed on a constant temperature shaker at 40 °C for 12 hours. The synthesized nanoparticles were dissolved in PBS (pH 7.4). Hydrodynamic size and zeta potential of the samples were measured using Zetasizer nano S90 (Malvern Instruments, UK) with dynamic light scattering (DLS) at room temperature.

**Degree of crosslinking**

A ninhydrin assay was performed to measure the amount of free amino groups of each samples using a commercially available kit (GenM3d, USA). The tested pericardial samples were lyophilized and heated with a ninhydrin solution for 20 minutes. The optical absorbance of the solutions was recorded with a spectrophotometer at 570 nm using glycine at various known concentrations as standard. Degree of crosslinking is calculated following the equation [5]:

Degree of crosslinking (%) = (1 – amine content in sample/amine content in non-crosslinked samaple) × 100%.

**Cell culture**

L929 fibroblasts were obtained from the American Type Culture Collection (ATCC) and cultured in Dulbecco’s modified Eagle’s medium (DMEM) (Gibco, UK) supplemented with 10% fetal bovine serum (FBS) (Gibco, UK), 100 U/ml penicillin (Gibco, UK) and 100 μg/ml streptomycin (Gibco, UK). Human umbilical vein endothelial cells (HUVECs) were cultured in endothelial cell medium (Gibco, UK) containing 10% FBS, 100 U/ml penicillin and 100 μg/ml streptomycin at 37°C in a humidified atmosphere of 5% CO_2_.

**Extract cytotoxicity assay**

Pericardial samples were finely minced, weighed, and incubated in indicated medium (1 mL medium per 100 mg tissue) for 72 hours with agitation. Following incubation, extract medium was centrifuged and supernatant was collected. Extract cytotoxicity was determined by CCK-8 assay (Dojindo, China) using L929 cells. According to the International standard ISO 10993-5 regarding tests for *in vitro* cytotoxicity of medical devices, materials leading to a cell viability result above 70% of the control were considered as non-cytotoxic.

**Hemolysis assay**

Hemolysis assay was performed using blood from donors. Briefly, red blood cells (RBCs) were isolated, washed and resuspended in PBS with the volume concentration of 2%. Samples (~1 cm^2^) were placed in the sterile eppendorf tube and one ml diluted RBC solution was added per tube and incubated at 37°C for 3 hours. The RBCs incubated in deionized water and PBS were used as the positive and negative controls, respectively. After incubation, the supernatant was collected after centrifugation, and the optical absorbance of the solutions was recorded with a spectrophotometer at 545 nm. The hemolysis rates were calculated using the following equation:

Hemolysis rate (%) = (*OD*_test_-*OD*_neg_) / (*OD*_pos_-*OD*_neg_) × 100%,

where *OD*_test_, *OD*_neg_, and *OD*_pos_ were the *OD*_545_ values of samples, negative control and positive control, respectively.

**Differential scanning calorimetry (DSC)**

DSC was used to measure the thermal denaturation temperature (Td) of the tested pericardial samples using a DSC 2500 Differential Scanning Calorimeter (TA Instruments, USA). Samples (6 ~10 mg) were blotted to remove surface water and placed in hermetically-sealed aluminum pans. Samples were heated from 20 to 90°C at a [heating rate of](https://www.sciencedirect.com/topics/engineering/rate-of-heating) 2 °C/minute. The resultant heating curves were analyzed using Thermal analysis software and the denaturation temperature was recorded at the height of the endothermic peak.

***In vitro* collagenase assay**

Samples were lyophilized, cut into pieces (~ 1 mm^3^) and weighed (initial dry weight). Then, the samples (40 mg) were treated with collagenase type I (1.5 mg/mL, Sigma Aldrich, Germany). At every predetermined time point, the samples were lyophilized and weighed again (final dry weight). The degree of enzymatic degradation of the samples was quantified as the percent weight loss (*W*%), which is calculated using the following formula [4,6]:

*W*% = (*W*_0_-*W*_t_) / *W*_0_× 100%,

Where *W*_0_ represents the initial weight of samples and *W*_t_ represents the weight of corresponding sample after enzymatic degradation treatment.

**Tensile testing**

Experiments were carried out using a Zwick tensile tester (Zwick GmbH & Co. KG). Pericardial samples of the same directions were cut into 50 (length) × 10 (width) mm rectangular strips. The mean thickness of each sample was determined by a series of measurements at four different points using a Mitutoyo digital micrometer. Samples were attached to grips. The tensile testing was performed at 5 mm/minute until failure. All testing was conducted at room temperature.

***In vitro* antibacterial activity assay**

Staphylococcus aureus (*S. aureus*, Gram positive bacteria) was cultured using nutrient agar medium (Difco™, USA). The density of *S. aureus* was adjusted to 10^6^ colony forming unit (CFU)/mL and bacteria suspension was inoculated on nutrient agar plates. Samples were cut into round pieces with a diameter of 15 mm and placed on the surface of agar plates, and co-cultured with *S. aureus* for 12 hours at 37 °C. The clear area indicating zone of inhibition was measured and recorded. The clear area indicating zone of inhibition was measured and recorded [7].

**Preparation of pericardial conditional medium**

The tested pericardial samples (~ 0.5 mm^3^/well) were placed in six-well plates and incubated in endothelial medium (2 mL) at 37 °C. The eluate medium from samples were collected at day 3 and stored at -80 °C until use.

***In vitro* tube formation assay**

Angiogenesis was assessed using HUVECs according to the previously reported procedure [8]. Matrigel® basement membrane matrix (Corning, USA) was thawed overnight at 4 °C and added to 96-well plates (50 µl/well) using cold tips on ice and incubated at 37 °C to solidify. HUVECs (3×10^4^ cells/well) were resuspended in eluate medium and seeded onto the solidified Matrigel-coated wells. After 12 hours of incubation, the wells were rinsed with PBS three times and then fixed with 4% polyformaldehyde, permeabilized with 0.2% Trition X-100 and stained with phalloidin (Servicebio, China) and DAPI (Sigma Aldrich, Germany) solution. Bright and fluorescence images were captured from each well using an inverted optical microscope (Olympus IX71, Japan). Data on total tubule length of capillary-like network were analyzed using ImageJ software.

**Subcutaneous implantation models**

The animal experiments were performed according to the “Guide for the Care and Use of Laboratory Animals” published by the National Institutes of Health (National Institutes of Health, 8^th^ Edition, 2010), all protocols were approved by the Institutional Animal Care and Use Committee of Changhai Hospital. Sprague Dawley rats (200 ~ 225 g) were obtained from the experimental Animal Center, Changhai Hospital (Shanghai, China). The rats were given at least one week for acclimatization. All animals had normal postoperative recovery, and none died or had complications during the study period.

To evaluate the *in vivo* responses including biostability, cell ingrowth and calcification, the tested samples were implanted subcutaneously into rats [4]. Rats were anesthetized, subcutaneous pockets were created on the dorsal side of each rat, and one small patch (a size of approximately 1 cm^2^) was inserted into each pocket. Subsequently, rats were allowed to recover and maintained in standard housing conditions with food and water ad libitum. At 21 days, rats were scarified and patch explants were harvested and then possessed for histological analysis and calcium quantitative analysis.

**Cardiac patch graft models**

To evaluate the capacity of integration with myocardial tissues, the tested samples were implanted to the heart of rats. Briefly, rats were anesthetized and heart was exposed through a median sternotomy. The samples were cut into round patches (~ 5mm) and laid on the top of cardiac wall and sutured to the margin of the patch with around ial tissues. The chest incision was closed with 5-0 Vicryl (Ethicon). No anticoagulation (or antiplatelets) therapy was administered. At 60 days, rats were scarified and hearts were harvested and then possessed for histological analysis. All animals had normal postoperative recovery, and none died or had complications during the study period.

**Histology**

The specimens were fixed in 4% buffered formaldehyde for 24 hours, processed into paraffin, and then sectioned at 5 μm. Sections were deparaffinized and stained with hematoxylin and eosin (HE) for morphological examination, with Verhoeff's Van Gieson (VG) staining for collagen and elastin, and with [alizarin](https://www.sciencedirect.com/topics/materials-science/alizarin) red S staining for detection of calcification. For Verhoeff's Van Gieson (VG) staining, sections were incubated in Verhoeff’s solution for 1 hour, rinsed in water, differentiated in 95 % ethanol, and incubated briefly in Van Gieson solution. For [alizarin](https://www.sciencedirect.com/topics/materials-science/alizarin) red S staining, deparaffinized tissue sections were incubated with 1% [alizarin](https://www.sciencedirect.com/topics/materials-science/alizarin) red S solution (pH 4.1). After incubation at room temperature for 20 minutes, the sections were washed again with deionized water to remove unincorporated dye.

**Immunohistochemistry**

The expression of α-smooth muscle actin (α-SMA) was evaluated using immunohistochemistry. In brief, sections were deparaffinized and hydrated to distilled water. Sections were then underwent antigen-retrieval in citrate buffer (0.01 M pH 6.0) at high temperature (water bath, 30 minutes at 98°C). After blocking for non-specific binding, α-SMA primary antibody (abcam, Cambridge, MA, dilution, 1:200) was applied at optimized concentrations and incubated overnight at 4 °C. Subsequently, they were incubated with secondary antibody and streptavidin-peroxidase complex at room temperature for 15 minutes each (SP kit, Fujian Maixin, China), and visualized with 3,3'-diaminobenzidine (DAB, Fujian Maixin, China).  Nuclear counterstaining was with haematoxylin.

**Calcium quantitative analysis**

Explants were weighed and decalcified in 6 N HCl at room temperature for 3 days. The calcium content of the 0.6 N HCl supernatant was determined colorimetrically using [alizarin](https://www.sciencedirect.com/topics/materials-science/alizarin) red S as indicator. The optical absorbance of the solutions was recorded with a spectrophotometer at 620 nm using calcium ion at various known concentrations as standard. The total calcium content of each explants was normalized to its dry weight.

**Copper ion quantitative analysis**

Copper ion concentration was determined by a colorimetric method using a copper analysis kit (Beijing Leagene Biotechnology Co., Ltd.). The optical absorbance of the solutions was recorded with a spectrophotometer (EPOCH, USA) at 620 nm using cuprizone as a colorimetric reagent.

**Statistical analysis**

Results are expressed as mean ± standard error (SD). Statistical analyses were performed using GraphPad Prism 6.0 (GraphPad Software, USA). For normal distributions, the differences between two of the groups were evaluated by means of the t-test, while one-way analysis of variance (ANOVA) for multiple samples. When the data distribution was not normally distributed, Manne Whitney or Krusal-Wallis non-parametric multiple-comparison test was employed. *P* < 0.05 was considered statistically significant.

**Reference**

1. [Xiaohong Liu](https://pubmed.ncbi.nlm.nih.gov/?term=Liu+X&cauthor_id=31693254), [Hao Wu](https://pubmed.ncbi.nlm.nih.gov/?term=Wu+H&cauthor_id=31693254), [Fanglin Lu](https://pubmed.ncbi.nlm.nih.gov/?term=Lu+F&cauthor_id=31693254), [Qin Li](https://pubmed.ncbi.nlm.nih.gov/?term=Li+Q&cauthor_id=31693254), [Zhiyun Xu](https://pubmed.ncbi.nlm.nih.gov/?term=Xu+Z&cauthor_id=31693254). Fabrication of porous bovine pericardium scaffolds incorporated with bFGF for tissue engineering applications. Xenotransplantation. 2020 Jan;27(1):e12568.

2. Vuong [QV](https://sci-hub.org.cn/extdomains/scholar.google.com.hk/citations?user=zK_Ki9cAAAAJ&hl=zh-TW&oi=sra), Golding [JB](https://sci-hub.org.cn/extdomains/scholar.google.com.hk/citations?user=ebT7JsAAAAAJ&hl=zh-TW&oi=sra), Nguyen [MH](https://sci-hub.org.cn/extdomains/scholar.google.com.hk/citations?user=0geqFDAAAAAJ&hl=zh-TW&oi=sra), Roach PD. Preparation of decaffeinated and high caffeine powders from green tea. Powder technology. 2013; 233:169-175.

3. [Tanur Sinha](https://pubmed.ncbi.nlm.nih.gov/?term=Sinha+T&cauthor_id=26300362), [M Ahmaruzzaman](https://pubmed.ncbi.nlm.nih.gov/?term=Ahmaruzzaman+M&cauthor_id=26300362). Green synthesis of copper nanoparticles for the efficient removal (degradation) of dye from aqueous phase. Environ Sci Pollut Res Int. 2015 Dec;22(24):20092-100.

4. [Amy E Munnelly](https://pubmed.ncbi.nlm.nih.gov/?term=Munnelly+AE&cauthor_id=21993239) [1](https://pubmed.ncbi.nlm.nih.gov/21993239/#affiliation-1), [Leonard Cochrane](https://pubmed.ncbi.nlm.nih.gov/?term=Cochrane+L&cauthor_id=21993239), [Joshua Leong](https://pubmed.ncbi.nlm.nih.gov/?term=Leong+J&cauthor_id=21993239), [Naren R Vyavahare](https://pubmed.ncbi.nlm.nih.gov/?term=Vyavahare+NR&cauthor_id=21993239). Porcine vena cava as an alternative to bovine pericardium in bioprosthetic percutaneous heart valves. Biomaterials. 2012 Jan;33(1):1-8.

5. [Malavika Nair](https://pubmed.ncbi.nlm.nih.gov/?term=Nair+M&cauthor_id=32480093), [Ramneek K Johal](https://pubmed.ncbi.nlm.nih.gov/?term=Johal+RK&cauthor_id=32480093), [Samir W Hamaia](https://pubmed.ncbi.nlm.nih.gov/?term=Hamaia+SW&cauthor_id=32480093), [Serena M Best](https://pubmed.ncbi.nlm.nih.gov/?term=Best+SM&cauthor_id=32480093), [Ruth E Cameron](https://pubmed.ncbi.nlm.nih.gov/?term=Cameron+RE&cauthor_id=32480093). Tunable bioactivity and mechanics of collagen-based tissue engineering constructs: A comparison of EDC-NHS, genipin and TG2 crosslinkers. Biomaterials. 2020 Sep;254:120109.

6. [Xiaoya Wang](https://pubmed.ncbi.nlm.nih.gov/?term=Wang+X&cauthor_id=25641644), [Wanyin Zhai](https://pubmed.ncbi.nlm.nih.gov/?term=Zhai+W&cauthor_id=25641644), [Chengtie Wu](https://pubmed.ncbi.nlm.nih.gov/?term=Wu+C&cauthor_id=25641644), [Bing Ma](https://pubmed.ncbi.nlm.nih.gov/?term=Ma+B&cauthor_id=25641644), [Jiamin Zhang](https://pubmed.ncbi.nlm.nih.gov/?term=Zhang+J&cauthor_id=25641644), [Hongfeng Zhang](https://pubmed.ncbi.nlm.nih.gov/?term=Zhang+H&cauthor_id=25641644), [Ziyan Zhu](https://pubmed.ncbi.nlm.nih.gov/?term=Zhu+Z&cauthor_id=25641644), [Jiang Chang](https://pubmed.ncbi.nlm.nih.gov/?term=Chang+J&cauthor_id=25641644). Procyanidins-crosslinked aortic elastin scaffolds with distinctive anti-calcification and biological properties. Acta Biomater. 2015 Apr;16:81-93.

7. [Mukesh Lavkush Bhaisare](https://pubmed.ncbi.nlm.nih.gov/?term=Bhaisare+ML&cauthor_id=26575840), [Bo-Sgum Wu](https://pubmed.ncbi.nlm.nih.gov/?term=Wu+BS&cauthor_id=26575840), [Mon-Chun Wu](https://pubmed.ncbi.nlm.nih.gov/?term=Wu+MC&cauthor_id=26575840), [M Shahnawaz Khan](https://pubmed.ncbi.nlm.nih.gov/?term=Khan+MS&cauthor_id=26575840), [Mei-Hwei Tseng](https://pubmed.ncbi.nlm.nih.gov/?term=Tseng+MH&cauthor_id=26575840), [Hui-Fen Wu](https://pubmed.ncbi.nlm.nih.gov/?term=Wu+HF&cauthor_id=26575840). MALDI MS analysis, disk diffusion and optical density measurements for the antimicrobial effect of zinc oxide nanorods integrated in graphene oxide nanostructures. Biomater Sci. 2016 Jan;4(1):183-94

8. [Emily J Ryan](https://pubmed.ncbi.nlm.nih.gov/?term=Ryan+EJ&cauthor_id=30708184), [Alan J Ryan](https://pubmed.ncbi.nlm.nih.gov/?term=Ryan+AJ&cauthor_id=30708184), [Arlyng González-Vázquez](https://pubmed.ncbi.nlm.nih.gov/?term=González-Vázquez+A&cauthor_id=30708184), [Anahí Philippart](https://pubmed.ncbi.nlm.nih.gov/?term=Philippart+A&cauthor_id=30708184), [Francesca E Ciraldo](https://pubmed.ncbi.nlm.nih.gov/?term=Ciraldo+FE&cauthor_id=30708184), [Christopher Hobbs](https://pubmed.ncbi.nlm.nih.gov/?term=Hobbs+C&cauthor_id=30708184), [Valeria Nicolosi](https://pubmed.ncbi.nlm.nih.gov/?term=Nicolosi+V&cauthor_id=30708184), [Aldo R Boccaccini](https://pubmed.ncbi.nlm.nih.gov/?term=Boccaccini+AR&cauthor_id=30708184), [Cathal J Kearney](https://pubmed.ncbi.nlm.nih.gov/?term=Kearney+CJ&cauthor_id=30708184), [Fergal J O'Brien](https://pubmed.ncbi.nlm.nih.gov/?term=O'Brien+FJ&cauthor_id=30708184). Collagen scaffolds functionalised with copper-eluting bioactive glass reduce infection and enhance osteogenesis and angiogenesis both in vitro and in vivo. Biomaterials. 2019 Mar;197:405-416.

**Supplementary Results**

**Supplementary** Table 1. Corresponding copper ion concentration in extract medium

| CuSO4 solution (mg/mL) | 0.1 | 0.2 | 0.3 | 0.4 | 0.5 | 0.6 | 0.7 | 0.8 | 0.9 | 1.0 |
| --- | --- | --- | --- | --- | --- | --- | --- | --- | --- | --- |
| Extract medium  (Cu^2+^ concentration, µg/mL) | 24.71 | 42.64 | 55.64 | 66.21 | 73.86 | 79.71 | 80.07 | 90.93 | 92.75 | 103.43 |


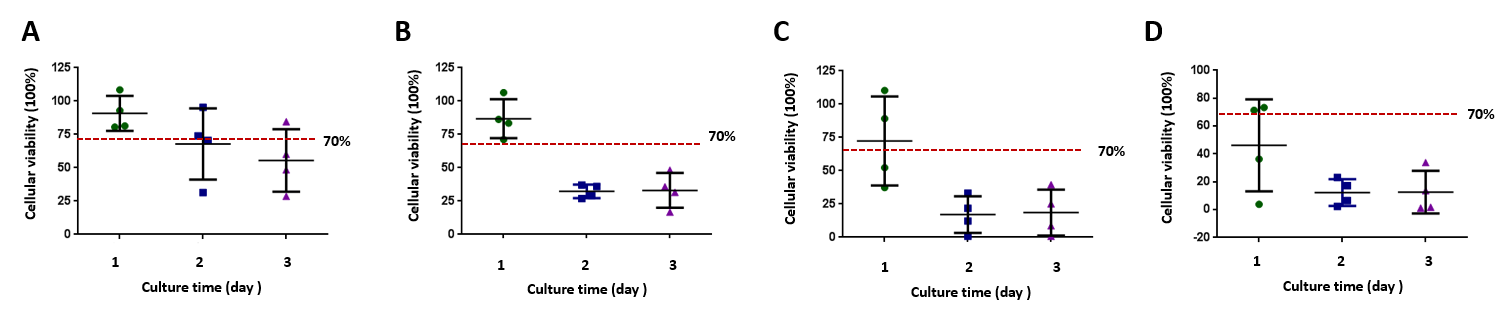


**Supplementary Figure 1. Cytotoxicity of Cu@TP-dBP extract medium on L929 cells.**

L929 cells were treated with extract medium of Cu@TP-dBPs fabricated using 0.2 (A), 0.3 (B), 0.4 (C) and 0.5 (D) mg/mL copper ions for three days. The red-dashed line represented the safety standard of 70% cell viability.


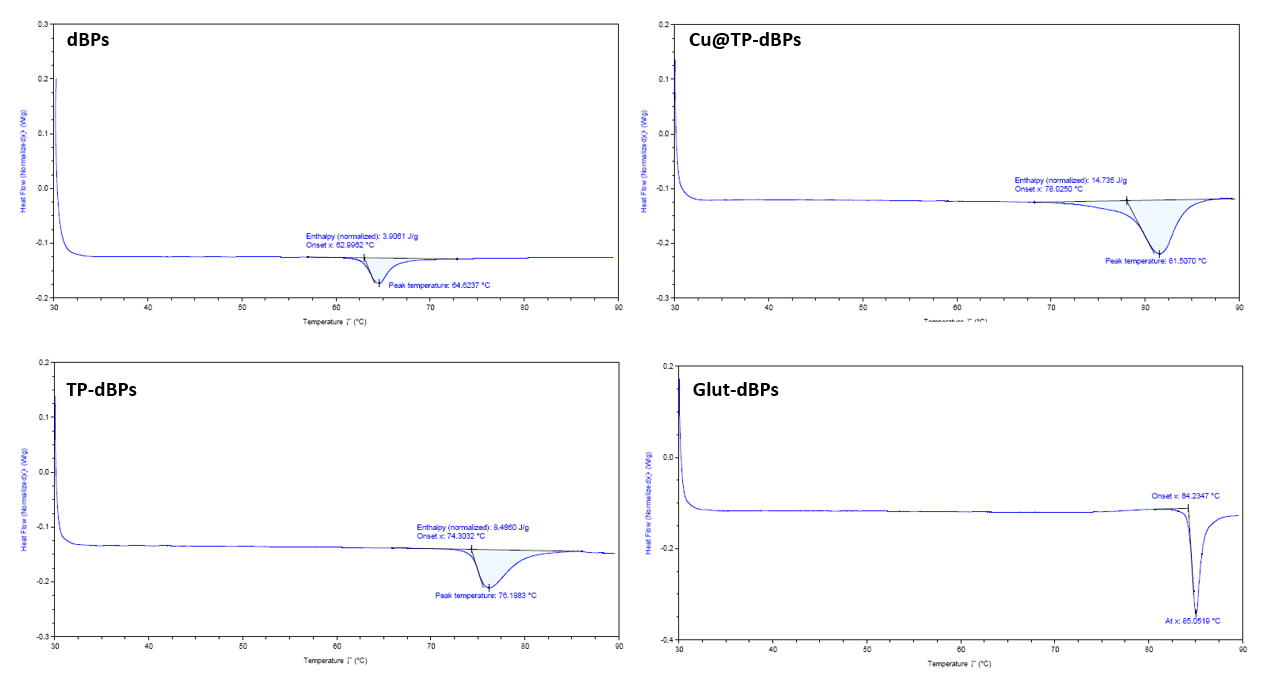


**Supplementary Figure 2. Collagen thermal stability of Cu@TP-dBPs assessed by DSC.**


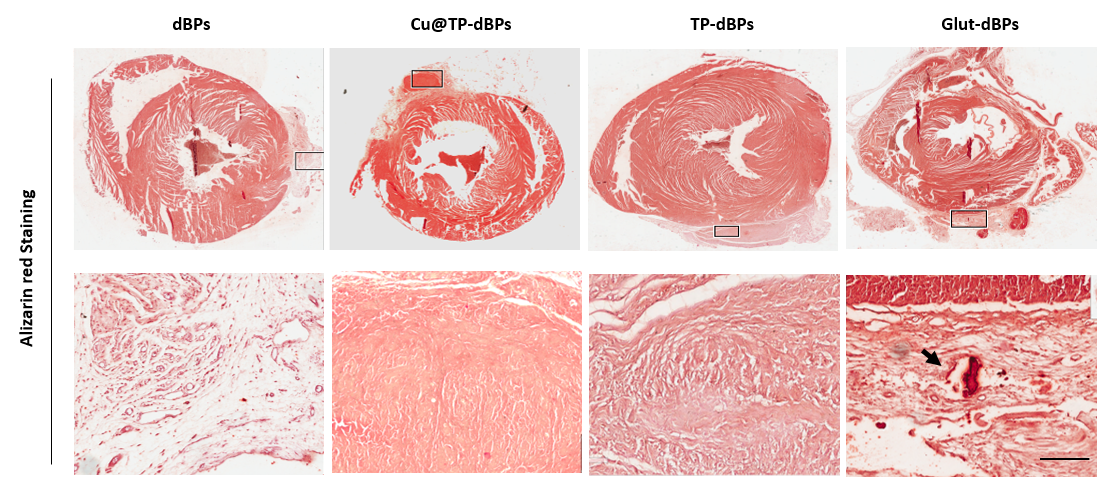


**Supplementary Figure 3. Calcification** o**f Cu@TP-dBPs in the rat cardiac patch graft models assessed by** [**alizarin**](https://www.sciencedirect.com/topics/materials-science/alizarin)**red S staining. Scale bar** = 150µm.


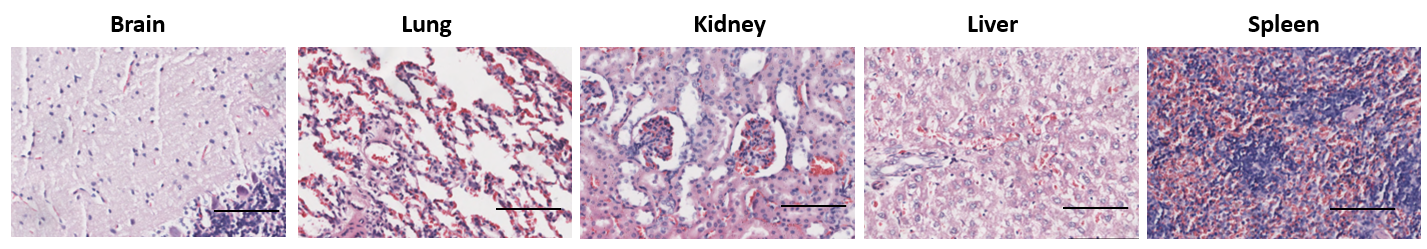


**Supplementary Figure 4. *In vivo* toxicity of Cu@TP-dBPs.** Brain, lung, kidney, liver and spleen were collected from the rat cardiac patch graft models and HE staining showed that no obvious pathological changes of these major organs. Scale bar = 100µm.
